# Supplementary material for: H+-Translocating Membrane-Bound Pyrophosphatase from Rhodospirillum rubrum Fuels Escherichia coli Cells via an Alternative Pathway for Energy Generation
Source: Microorganisms. 2023 Jan 23;11(2):294. doi: 10.3390/microorganisms11020294 (PMC9959109; doi:10.3390/microorganisms11020294)
Supplement: Supplementary file 1 [file microorganisms-11-00294-s001.zip › microorganisms-2075103-supplementary.pdf]

Supplementary Materials

Table S1. Metabolic map of *E. coli* central metabolism used for ^{13}C-MFA.

Pathway	No.	Reaction	Rearrangement of carbon atoms	Type of reaction*
Input	1	glc_D_e = glc_D_c	abcdef = abcdef	F
PTS transport	2	glc_D_c + pep_c = g6p_c + pyr_c	abcdef + ghi = abcdef + ghi	F
EMP	3	g6p_c = f6p_c	abcdef = abcdef	FR
	4	f6p_c = g6p_c	abcdef = abcdef	R
	5	f6p_c + ATP = fdp_c	abcdef + X = abcdef	F
	6	fdp_c = f6p_c	abcdef = abcdef	F
	7	fdp_c = dhap_c + g3p_c	abcdef = abc + def	FR
	8	dhap_c + g3p_c = fdp_c	abc + def = abcdef	R
	9	dhap_c = g3p_c	abc = cba	FR
	10	g3p_c = dhap_c	cba = abc	R
	11	g3p_c = 13dpg_c + NADH	abc = abc + X	FR
	12	13dpg_c + NADH = g3p_c	abc + X = abc	R
	13	13dpg_c = 3pg_c + ATP	abc = abc + X	FR
	14	3pg_c + ATP = 13dpg_c	abc + X = abc	R
	15	3pg_c = pep_c	abc = abc	FR
	16	pep_c = 3pg_c	abc = abc	R
	17	pep_c = pyr_c + ATP	abc = abc + X	F
	18	pyr_c + ATP + ATP = pep_c	abc + X + X = abc	F
PP	19	g6p_c = 6pgc_c + NADPH	abcdef = abcdef + X	F
	20	6pgc_c = co2_c + ru5p_D_c + NADPH	abcdef = a + bcdef + X	F
	21	ru5p_D_c = xu5p_D_c	abcde = abcde	FR
	22	xu5p_D_c = ru5p_D_c	abcde = abcde	R
	23	ru5p_D_c = r5p_c	abcde = abcde	FR
	24	r5p_c = ru5p_D_c	abcde = abcde	R
	25	xu5p_D_c = c2 + g3p_c	abcde = ab + cde	FR
	26	c2 + g3p_c = xu5p_D_c	ab + cde = abcde	R
	27	f6p_c = c2 + e4p_c	abcdef = ab + cdef	FR
	28	c2 + e4p_c = f6p_c	ab + cdef = abcdef	R
	29	s7p_c = c2 + r5p_c	abcdefg = ab + cdefg	FR
	30	c2 + r5p_c = s7p_c	ab + cdefg = abcdefg	R
	31	f6p_c = c3 + g3p_c	abcdef = abc + def	FR
	32	c3 + g3p_c = f6p_c	abc + def = abcdef	R
	33	s7p_c = c3 + e4p_c	abcdefg = abc + defg	FR
	34	c3 + e4p_c = s7p_c	abc + defg = abcdefg	R
ED	35	6pgc_c = 2ddg6p_c	abcdef = abcdef	F
	36	2ddg6p_c = g3p_c + pyr_c	abcdef = def + abc	F
PRPP synthesis	37	r5p_c + ATP + ATP = prpp_c	abcde + X + X = abcde	F
PDH	38	pyr_c = accoa_c + co2_c + NADH	abc = bc + a + X	F
TCA	39	accoa_c + oaa_c = cit_c	ab + cdef = fedbac	F
	40	cit_c = icit_c	abcdef = abcdef	FR
	41	icit_c = cit_c	abcdef = abcdef	R
	42	icit_c = akg_c + co2_c + NADPH	abcdef = abcde + f + X	FR
	43	akg_c + co2_c + NADPH = icit_c	abcde + f + X = abcdef	R

Pathway	No.	Reaction	Rearrangement of carbon atoms	Type of reaction*
	44	akg_c = co2_c + succoa_c + NADH	abcde = a + bcde + X	F
	45	succoa_c = succ_c + ATP	abcd = abcd + X	FR
	46	succ_c + ATP = succoa_c	abcd + X = abcd	R
	47	succ_c = 0.5fum + 0.5fum + FADH	abcd = 0.5abcd + 0.5dcba + X	FR
	48	fum + FADH = 0.5succ_c + 0.5succ_c	abcd + X = 0.5abcd + 0.5dcba	R
	49	fum = mal_L_c	abcd = abcd	FR
	50	mal_L_c = 0.5fum + 0.5fum	abcd = 0.5abcd + 0.5dcba	R
	51	mal_L_c = oaa_c + NADH	abcd = abcd + X	FR
	52	oaa_c + NADH = mal_L_c	abcd + X = abcd	R
GLX shunt	53	icit_c = glx_c + 0.5succ_c + 0.5succ_c	abcdef = ab + 0.5cdef + 0.5fedc	F
	54	accoa_c + glx_c = mal_L_c	ab + cd = cdba	F
malic enzyme	55	mal_L_c = co2_c + pyr_c + NADH	abcd = d + abc + X	F
	56	mal_L_c = co2_c + pyr_c + NADPH	abcd = d + abc + X	F
PCK	57	oaa_c + ATP = co2_c + pep_c	abcd + X = d + abc	F
PPC	58	co2_c + pep_c = oaa_c	d + abc = abcd	F
Acetate synthesis and excretion	59	accoa_c = ac_c + ATP	ab = ab + X	FR
	60	ac_c + ATP = accoa_c	ab + X = ab	R
	61	pyr_c + q8 = ac_c + co2_c + q8h2	abc + X = bc + a + X	F
	62	ac_c = ac_ex	ab = ab	F
CO2 excretion	63	co2_c = co2_ex	a = a	F
CO2 exchange	64	co2_nat + co2_c = co2_c + co2_nat	a + b = a + b	F
C1 metabolism	65	mLthf_c = 10fthf_c + NADPH	a = a + X	F
	66	mLthf_c + NADH = 5mthf_c	a + X = a	F
Glu synthesis	67	akg_c + NADPH + NH3 = glu_L_c	abcde + X + X = abcde	F
Asp synthesis	68	glu_L_c + oaa_c = akg_c + asp_L_c	abcde + fghi = abcde + fghi	FR
	69	akg_c + asp_L_c = glu_L_c + oaa_c	abcde + fghi = abcde + fghi	R
Ala synthesis	70	glu_L_c + pyr_c = akg_c + ala_L_c	abcde + fgh = abcde + fgh	F
Gln synthesis	71	glu_L_c + ATP + NH3 = gln_L_c	abcde + X + X = abcde	F
Ser synthesis	72	3pg_c + glu_L_c = akg_c + pser_L_c + NADH	abc + defgh = defgh + abc + X	F
	73	pser_L_c = ser_L_c	abc = abc	F
Ser degradation	74	ser_L_c = pyr_c + NH3	abc = abc + X	F
Gly synthesis	75	ser_L_c = gly_c + mLthf_c	abc = ab + c	FR
	76	gly_c + mLthf_c = ser_L_c	ab + c = abc	R
	77	thr_L_c = accoa_c + gly_c + NADH	abcd = cd + ab + X	F
Gly cleavage	78	gly_c = co2_c + mLthf_c + NADH + NH3	ab = a + b + X + X	F
Val synthesis	79	pyr_c + pyr_c = alac_S_c + co2_c	abc + def = abcef + d	F
	80	alac_S_c + NADPH = 3mob_c	abcde + X = abdce	F
	81	3mob_c + glu_L_c = akg_c + val_L_c	abcde + fghij = fghij + abcde	F
Leu synthesis	82	3mob_c + accoa_c = 3c3hmp_c	abcde + fg = abfgcde	F
	83	3c3hmp_c = 2ippc	abcdefg = abcdefg	F
	84	2ippc = 3c2hmp_c	abcdefg = abcdefg	F
	85	3c2hmp_c = 3c4mop_c + NADH	abcdefg = abcdefg + X	F
	86	3c4mop_c = 4mop_c + co2_c	abcdefg = bcdefg + a	F

Pathway	No.	Reaction	Rearrangement of carbon atoms	Type of reaction*
	87	4mop_c + glu_L_c = akg_c + leu_L_c	abcdef + ghijk = ghijk + abcdef	F
Ile synthesis	88	thr_L_c = 2obut_c + NH3	abcd = abcd + X	F
	89	2obut_c + pyr_c = 2ahbut_c + co2_c	abcd + efg = abcdfg + e	F
	90	2ahbut_c + NADPH = 23dhmp_c	abcdef + X = abecdf	F
	91	23dhmp_c = 3mop_c	abcdef = abcdef	F
	92	3mop_c + glu_L_c = akg_c + ile_L_c	fghijk + abcde = abcde + fghijk	F
Phe, Tyr, Trp synthesis	93	e4p_c + pep_c = 2dda7p_c	abcd + efg = efgabcd	F
	94	2dda7p_c + NADPH = skm_c	abcdefg + X = bcdefga	F
	95	skm_c + ATP = skm5p_c	abcdefg + X = abcdefg	F
	96	pep_c + skm5p_c = 3psme_c	hij + abcdefg = abcdefhijg	F
	97	3psme_c = chor_c	abcdefghij = abcdefghij	F
	98	chor_c = pphn_c	abcdefghij = abcdefghij	F
	99	pphn_c = co2_c + phpyr_c	abcdefghij = j + ghiabcdef	F
	100	glu_L_c + phpyr_c = akg_c + phe_L_c	abcde + fghijklmn = abcde + fghijklmn	F
	101	pphn_c = 34hpp_c + co2_c + NADH	abcdefghij = ghiabcdef + j + X	F
	102	34hpp_c + glu_L_c = akg_c + tyr_L_c	fghijklmn + abcde = abcde + fghijklmn	F
	103	chor_c + gln_L_c = anth_c + glu_L_c + pyr_c	abcdefghij + klmno = abcdefj + klmno + ghi	F
	104	anth_c + prpp_c = pran_c	abcdefg + hijkl = abcdefghijkl	F
	105	pran_c = 2cpr5p_c	abcdefghijkl = hijklbafedcg	F
	106	2cpr5p_c = 3ig3p_c + co2_c	abcdefghijkl = edcabghijkf + l	F
	107	3ig3p_c = g3p_c + indole_c	abcdefghijk = cba + defghijk	F
	108	indole_c + ser_L_c = trp_L_c	defghijk + abc = abcdefghijk	F
Cys synthesis	109	accoa_c + ser_L_c = acser_c	ab + cde = cdeab	F
	110	acser_c + h2s = ac_c + cys_L_c	abcde + X = de + abc	F
Met synthesis	111	hoL_c + succoa_c = suchms_c	abcd + efgh = abcdefgh	F
	112	cys_L_c + suchms_c = cyst_L_c + 0.5succ_c + 0.5succ_c	ijk + abcdefgh = abcdkji + 0.5efgh + 0.5hgfe	F
	113	cyst_L_c = hcys_L_c + pyr_c + NH3	abcdefg = abcd + gfe + X	F
	114	5mthf_c + hcys_L_c = met_L_c	e + abcd = abcde	F
Lys synthesis	115	asp_L_c + ATP + NADPH = aspsa_c	abcd + X + X = abcd	F
	116	aspsa_c + pyr_c = 23dhdp_c	abcd + efg = abcdgfe	F
	117	23dhdp_c + NADPH = thdp_c	abcdefg + X = abcdefg	F
	118	succoa_c + thdp_c = sl2a6o_c	hijk + abcdefg = abcdefghijk	F
	119	glu_L_c + sl2a6o_c = akg_c + sl26da_c	lmnop + abcdefghijk = lmnop + abcdefghijk	F
	120	sl26da_c = 26dap_LL_c + 0.5succ_c + 0.5succ_c	abcdefghijk = abcdefg + 0.5hijk + 0.5kjih	F
	121	26dap_LL_c = 0.526dap_c + 0.526dap_c	abcdefg = 0.5abcdefg + 0.5gfedcba	F
	122	26dap_c = co2_c + lys_L_c	abcdefg = g + abcdef	F
Pro synthesis	123	glu_L_c + ATP + NADPH + NADPH = pro_L_c	abcde + X + X + X = abcde	F
Arg synthesis	124	accoa_c + glu_L_c = acglu_c	ab + cdefg = cdefgab	F
	125	acglu_c + ATP = acg5p_c	abcdefg + X = abcdefg	F
	126	acg5p_c + NADPH = acg5sa_c	abcdefg + X = abcdefg	F
	127	acg5sa_c + glu_L_c = acorn_c + akg_c	abcdefg + hijkl = abcdefg + hijkl	F
	128	acorn_c = ac_c + orn_c	abcdefg = fg + abcde	F
	129	co2_c + gln_L_c + ATP + ATP = cbp_c + glu_L_c	a + bcdef + X + X = a + bcdef	F
	130	cbp_c + orn_c = citr_L_c	f + abcde = abcdef	F

Pathway	No.	Reaction	Rearrangement of carbon atoms	Type of reaction*
	131	asp_L_c + citr_L_c + ATP + ATP = argsuc_c	abcd + efghij + X + X = efghijabcd	F
	132	argsuc_c = arg_L_c + 0.5fum + 0.5fum	abcdefghij = abcdef + 0.5ghij + 0.5jihg	F
Thr synthesis	133	aspsa_c + NADPH = hoL_c	abcd + X = abcd	F
	134	hoL_c + ATP = thr_L_c	abcd + X = abcd	F
Asn synthesis	135	asp_L_c + ATP + ATP + NH3 = asn_L_c	abcd + X + X + X = abcd	F
His synthesis	136	atp_c + prpp_c = prbatp_c + aicar_c	f + abcde = abcdef + X	F
	137	prbatp_c = prlp_c	abcdef = abcdef	F
	138	gln_L_c + prlp_c = eig3p_c + glu_L_c	abcde + fghijk = fghijk + abcde	F
	139	eig3p_c = imacp_c	abcdef = abcdef	F
	140	glu_L_c + imacp_c = akg_c + hisp_c	abcde + fghijk = abcde + fghijk	F
	141	hisp_c = his_L_c + 2NADH	abcdef = abcdef + X	F
UTP, CTP	142	asp_L_c + cbp_c + q8 = orot_c + q8h2	abcd + e + X = abcde + X	F
	143	orot_c + prpp_c + ATP = udp_c + co2_c	abcde + X + X = bcde + a	F
	144	udp_c + ATP = utp_c		B
	145	utp_c + gln_L_c + ATP = ctp_c + glu_L_c	X + abcde + X = X + abcde	F
IMP	146	gln_L_c + prpp_c = glu_L_c + pram_c	abcde + X = abcde + X	F
	147	pram_c + gly_c + ATP = gar_c		B
	148	gar_c + 10fthf_c + ATP = fgam_c		B
	149	fgam_c + gln_L_c + ATP = fpram_c + glu_L_c	X + abcde + X = X + abcde	F
	150	fpram_c + co2_c + 2ATP = 5caiz_c		B
	151	5caiz_c + asp_L_c + ATP = 25aics_c	X + abcd + X = abcd	F
	152	25aics_c = aicar_c + 0.5fum + 0.5fum	abcd = X + 0.5abcd + 0.5dcba	F
	153	aicar_c + 10fthf_c = imp	X + a = a	F
ATP	154	asp_L_c + imp + ATP = dcamp_c	abcd + e + X = abcde	F
	155	dcamp_c + ATP = adp_c + 0.5fum + 0.5fum	abcde + X = e + 0.5abcd + 0.5dcba	F
	156	adp_c + 0.5NADH + 0.25O2 = atp_c	a + X + X = a	F
GTP	157	gln_L_c + imp + 4ATP = glu_L_c + gtp_c + NADH	abcde + X + X = abcde + X + X	F
dNTP (*via* thioredoxine)	158	ctp_c + NADPH = dctp_c		B
	159	atp_c + NADPH = datp_c		B
	160	gtp_c + NADPH = dgtp_c		B
	161	udp_c + NADPH = dump_c		B
	162	utp_c + NADPH = dump_c		B
dTTP	163	dump_c + mLthf_c + 2ATP = dttp_c		B
Biomass	164	biomass		B
Not carbon exchange reactions	169	so4_e + ATP = so4		**
	170	so4 + 3ATP + 4NADPH = h2s		
	171	NH3_ex = NH3		
	172	O2_ex = O2		
	173	ATP = ATP_ex		
	174	NADH = NADPH		
	175	2NADH + O2 = 4ATP		
	176	2FADH + O2 = 2ATP		

* F – irreversible reaction, FR – forward branch of a reversible reaction, R – backward branch of a reversible reaction, B – reactions that are taken into account at carbon

balancing, but not at isotopomer balancing (typically reactions that drain biomass precursors).

** The reactions Nos. 169-176 were excluded from the model used for flux calculation, as they do not relate to carbon or isotopomer balancing.

Table S2. Biomass composition of *E. coli* strains.

Biomass component	MG1655	MG1655 *IS*5.8::P_L-*hppa*^{Rru}	MG1655 *IS*5.8::P_L-*hppa*^{Rru} Δ*ppa*::*cat*	MG1655 *IS*5.8::P_L-*hppa*^{Rru} Δ*ppa*::*cat* ($K_{DW/OD}$ = 0.51)
Protein, g/gDW	0.55*	0.55*	0.45*	0.6
RNA, g/gDW	0.12*	0.12*	0.08*	0.1
DNA, g/gDW	0.03	0.03	0.03	0.03
Phospholipids, g/gDW	0.16	0.16	0.23	0.14
Lipopolysaccharides, g/gDW	0.06	0.06	0.09	0.05
Peptydoglycan, g/gDW	0.04	0.04	0.06	0.04
Glycogen, g/gDW	0.04	0.04	0.06	0.04

* The values were determined experimentally.

Table S3. Growth characteristics of *E. coli* MG1655, MG1655 *IS*5.8::P_L-*hppa*^{Rru}, and MG1655 *IS*5.8::P_L-*hppa*^{Rru} Δ*ppa*::*cat* strains under batch cultivation conditions.

Parameter	MG1655	MG1655 *IS*5.8::P_L-*hppa*^{Rru}	MG1655 *IS*5.8::P_L-*hppa*^{Rru} Δ*ppa*::*cat*
μ, 1/hour	0.64 ± 0.07	0.59 ± 0.06	0.23 ± 0.02
Biomass yield, gDW/g	0.40 ± 0.01	0.41 ± 0.01	0.30 ± 0.01
q glucose, mmol/gDW*hour	9 ± 1	8.2 ± 0.5	4.3 ± 0.2
q CO_2, mmol/gDW*hour	17 ± 4	18 ± 3	12 ± 3
q Acetate, mmol/gDW*hour	3.3 ± 0.1	3.1 ± 0.1	3.2 ± 0.7
K_{595}, mg DW in 1 mL at OD_{595} of 1	0.51 ± 0.03	0.52 ± 0.02	0.68 ± 0.02

C-mol yield biomass, %	48 ± 2	50 ± 2	37 ± 3
C-mol yield CO_2, %	33 ± 2	36 ± 4	46 ± 11
C-mol yield acetate, %	13 ± 2	12.5 ± 0.2	24 ± 5
C-mol balance, %	93 ± 2	98 ± 2	108 ± 14

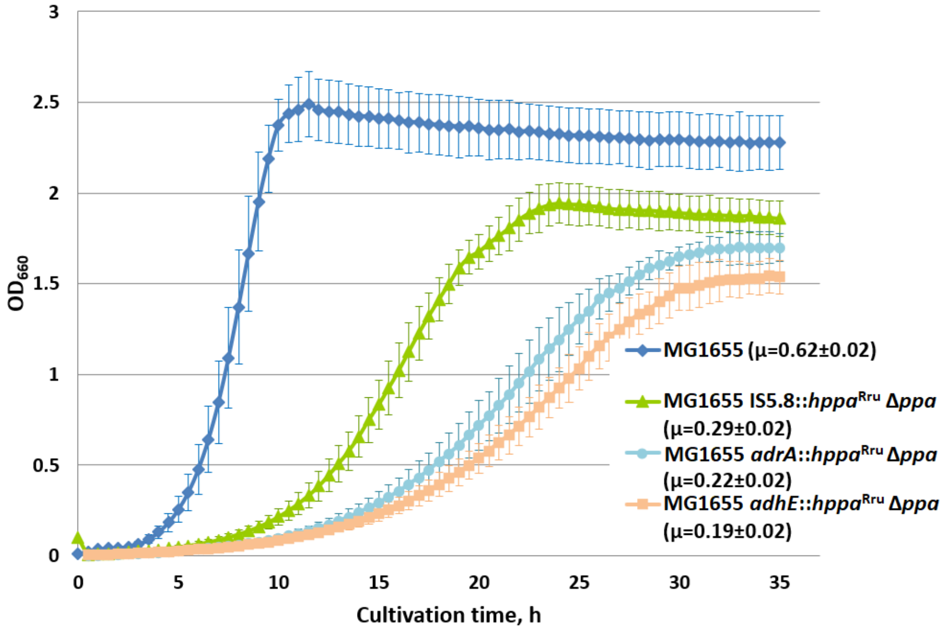

Figure S1. Growth of strains with the *hppa*^{Rru} gene in the presence and absence of *E. coli ppa* gene. Cells were grown with aeration in L-tubes with M9 minimal medium supplemented with 0.3% of glucose at 37 °C in Advantec photorecorder. MG1655, MG1655 wild-type; MG1655- *IS*5.8::*hppa*^{Rru} Δ*ppa*, MG1655 *IS*5.8::P$_L$-*hppa*^{Rru} Δ*ppa::cat*; MG1655 *adrA*::*hppa* ^{Rru} Δ*ppa*, MG1655 *adrA*::P$_L$-*hppa*^{Rru} Δ*ppa::cat*; MG1655 *adhE*::*hppa* ^{Rru} Δ*ppa*, MG1655 *adhE*::P$_L$-*hppa*^{Rru} Δ*ppa::cat*. Average data are presented; bars refer to standard deviations obtained in three independent experiments.

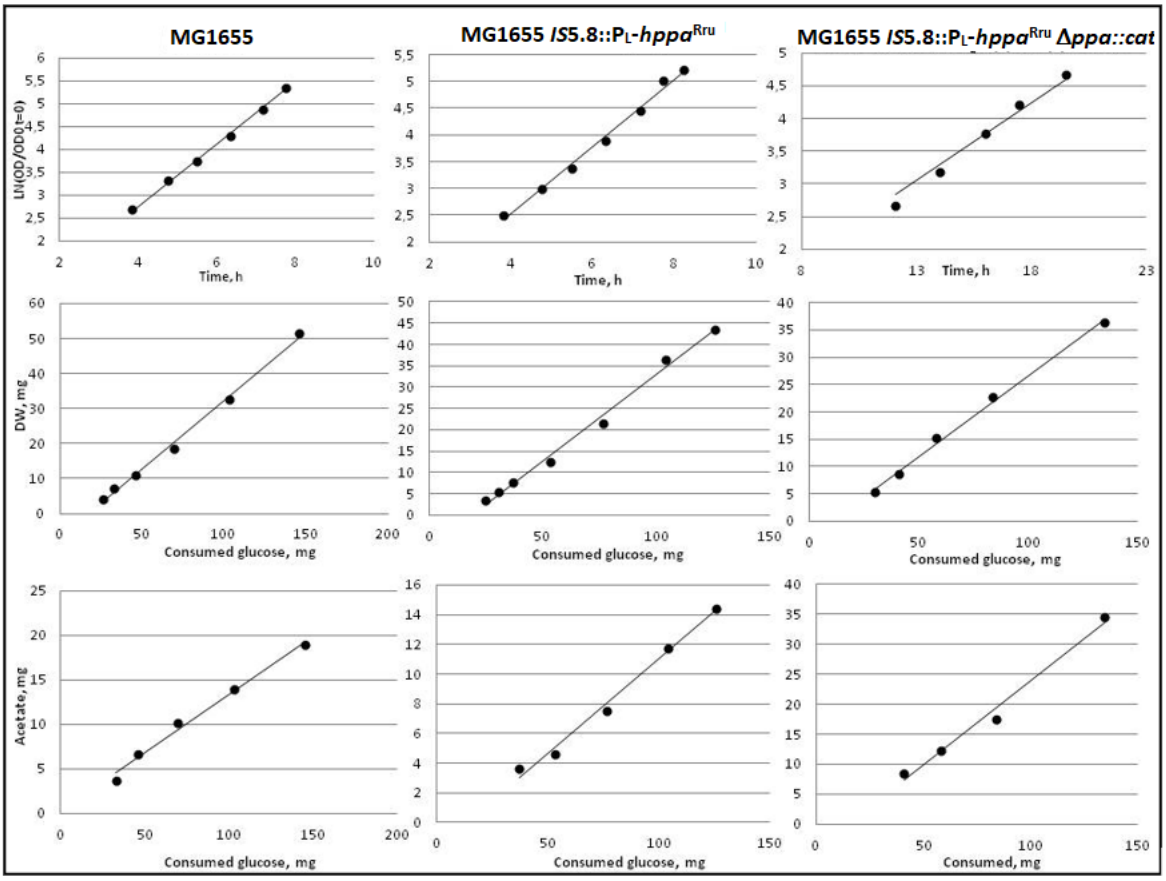

Figure S2. Cultivation profiles of *E. coli* strains MG1655, MG1655 *IS*5.8::P_L-*hppa*^{Rru} and MG1655 *IS*5.8::P_L-*hppa*^{Rru} Δ*ppa*::*cat* in batch culture with glucose. The metabolic steady-state during cultivation was confirmed by (i) the exponential mode of growth; (ii) linear correlation between production of biomass and glucose consumption; (iii) linear correlation between acetate production and consumption of glucose.

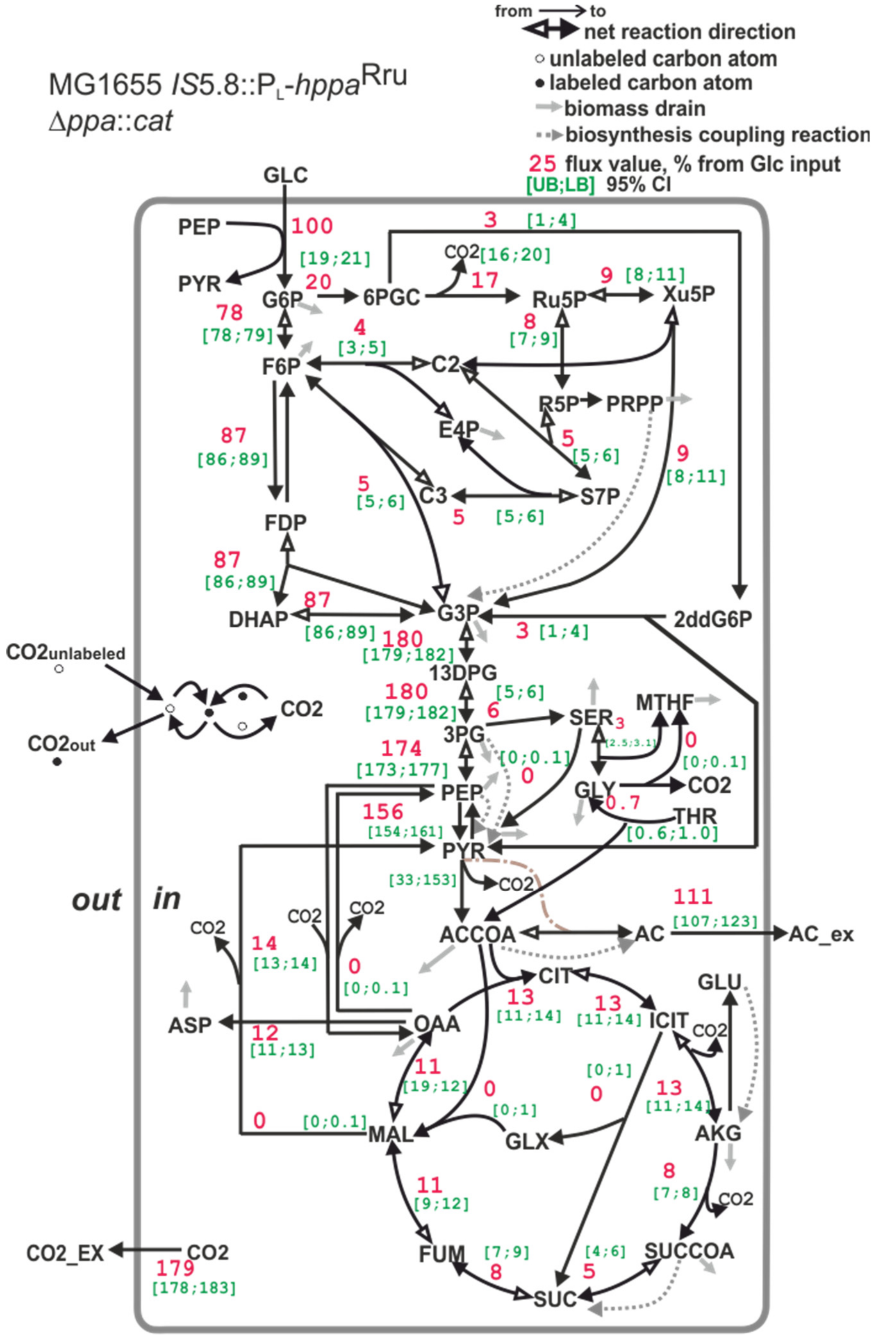

Figure S3. The carbon flux distribution in *E. coli* MG1655 *IS*5.8::P$_L$-*hppa*^{Rru} Δ*ppa*::Cm^R strain under assumption of the $K_{DW/OD}$ = 0.51. Flux through the PEP->PYR reaction is a sum of PTS-dependent glucose transport reaction and pyruvate kinase reaction. Flux through the pyruvate dehydrogenase reaction is expressed as a range (see Section 2.9.3.) Accounting for serine degradation to pyruvate (activated, for example, in an *E. coli pfkA*-deficient mutant, see [82]) leads to a broad confidence interval of lumped fluxes of phosphoglycerate mutase and enolase, and of pyruvate kinase flux.

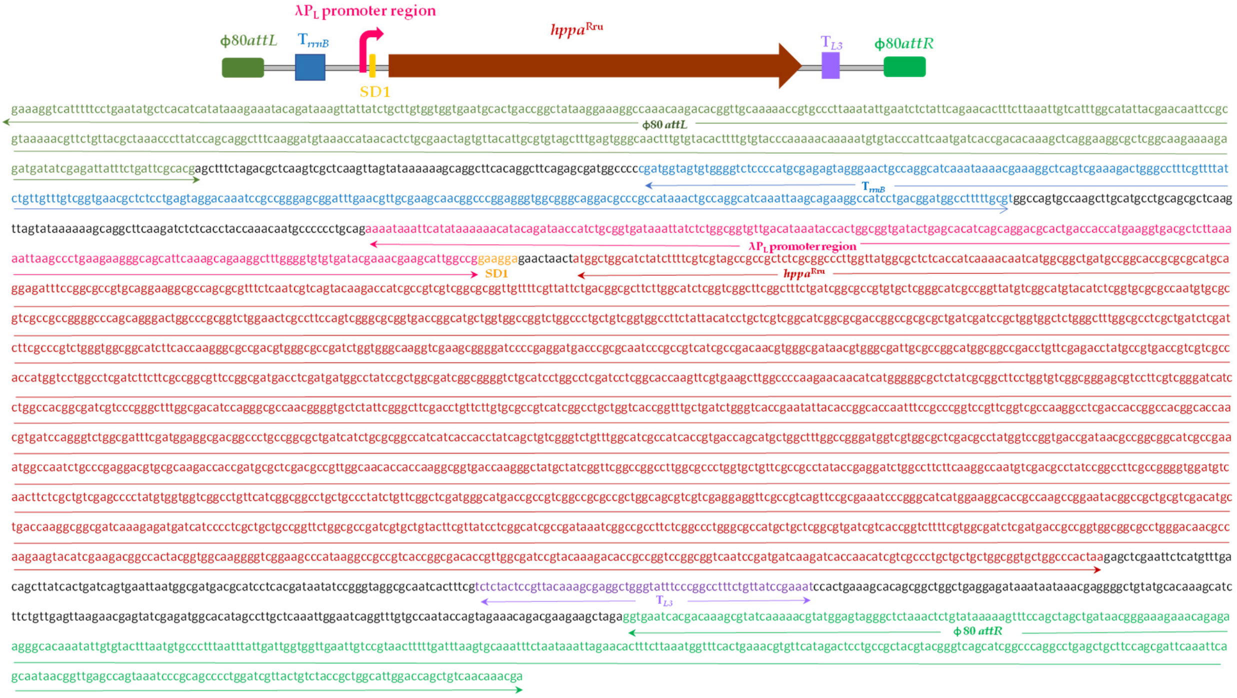

Figure S4. Schematic map of PL-*hppa*Rru expression cassette. The *hppa*Rru gene and main regulatory elements are shown, including sequence annotation: ϕ80*attL* and ϕ80*attR* are two hybrid sites, formed on the left and right sides as a result of ϕ80-mediated integration [2; 3]; T*rrnB*, terminator region of *E. coli* ribosomal operon *rrnB*; λPL promoter region, a promoter region from phage λ; SD1, Shain-Dalgarno sequence; *hppa*Rru, codon-optimized sequence of *R. rubrum hppa* gene; TL3, phage λ terminator.

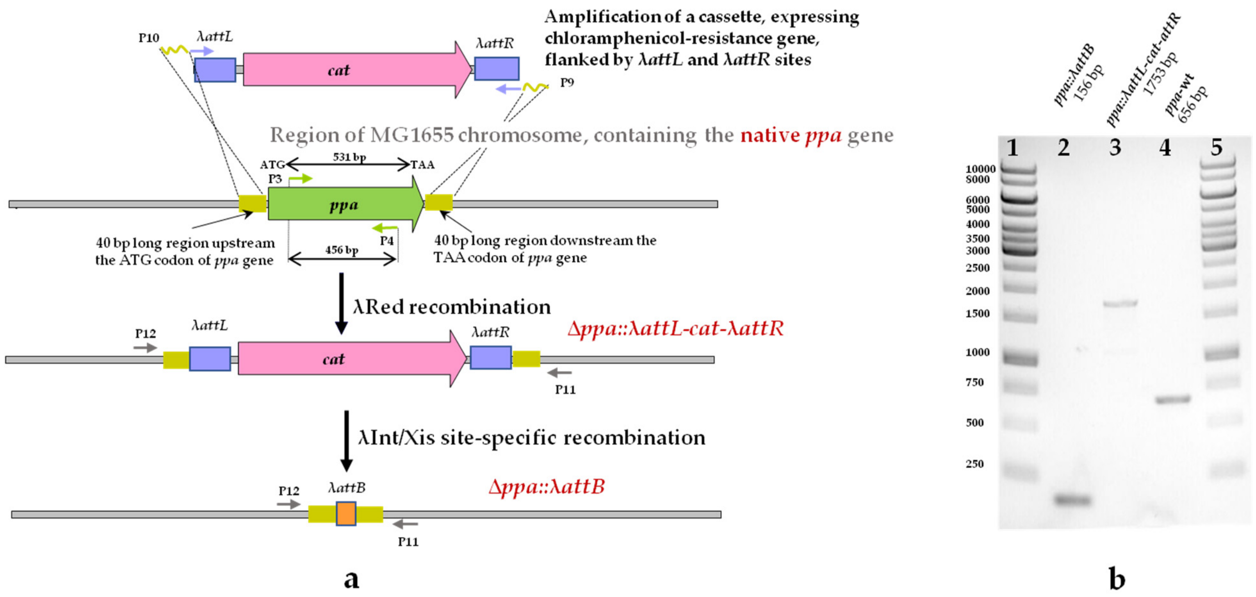

Figure S5. Construction of the essential *ppa* gene deletion in *E. coli* chromosome. (a) The scheme shows all steps of construction including (b) verification of *ppa* gene deletion by PCR analysis. P9, P10 – primers for amplification of the fragment for the *ppa* gene deletion; P11, P12 – primers for verification of *ppa* gene deletion. Lanes 1 and 5, DNA molecular weight Marker (1 kb Ladder); lane 2, PCR fragment of the marker-less *ppa* gene deletion, Δ*ppa*::λ*attB* (156 bp); lane 3, PCR fragment of *ppa* gene deletion marked by chloramphenicol resistance marker, Δ*ppa*::λ*attL-cat-*λ*attR* (1753 bp); lane 4, PCR fragment of the wild-type *ppa* gene. The primers P3

and P4, which were used to prepare the biotin-labeled probes for Southern-blot analysis, are also indicated.
